# SpeCond: a method to detect condition-specific gene expression

**DOI:** 10.1186/gb-2011-12-10-r101

**Published:** 2011-10-18

**Authors:** Florence MG Cavalli, Richard Bourgon, Wolfgang Huber, Juan M Vaquerizas, Nicholas M Luscombe

**Affiliations:** 1 EMBL-European Bioinformatics Institute, Wellcome Trust Genome Campus, Cambridge CB10 1SD, UK; 2 Department of Bioinformatics, Genentech Inc., 1 DNA Way, South San Francisco, CA 94080, USA; 3 EMBL-Heidelberg Genome Biology Unit, Meyerhofstraße 1, 69117 Heidelberg, Germany

## Abstract

Transcriptomic studies routinely measure expression levels across numerous conditions. These datasets allow identification of genes that are specifically expressed in a small number of conditions. However, there are currently no statistically robust methods for identifying such genes. Here we present SpeCond, a method to detect condition-specific genes that outperforms alternative approaches. We apply the method to a dataset of 32 human tissues to determine 2,673 specifically expressed genes. An implementation of SpeCond is freely available as a Bioconductor package at http://www.bioconductor.org/packages/release/bioc/html/SpeCond.html.

## Background

Cells sharing the same genomic information are able to express it in different ways to achieve cell-specific functions or respond to different environmental changes. Transcriptional regulation is the first step at which this specificity is determined, as it is the most basic level at which gene expression is controlled. Recent surveys of transcriptomic data across numerous cell types revealed two broad categories of gene expression: ubiquitous; and tissue- or cell-type-specific expression [1,2]. The first category contains genes that are expressed in most tissues at similar levels and they are thought to provide core cellular functionality [3,4]. The second category comprises genes with distinct expression in a few tissues or conditions, which are likely to be important for defining cell-specific functions.

In datasets with only a few conditions, it is possible to compare pairs of conditions using standard or moderated *t*-tests [5-7]. However, this becomes impractical with large datasets, as the number of pairwise comparisons increases exponentially with respect to the number of conditions studied. An alternative method is the non-standard ANOVA, which tests all possible groups of samples against each other. However, this involves computationally intensive dynamic programming and cannot detect specificity in individual conditions. Moreover, the method requires equal standard deviations between all groups of conditions being compared: this cannot be assumed as genes might have similar expression levels in some conditions - and thus small standard deviations - and more divergent expression levels in others. A further alternative is the Tukey test, although this method requires independence between groups of conditions and a normal distribution of group means, criteria that are often not met in microarray experiments. Importantly, most of these and other methods assume that expression values follow a single normal distribution. This assumption is generally not satisfied, which means that methods do not model the data correctly and therefore lead to false positive results [8].

An alternative to these approaches is a mixture model-based procedure to model gene expression. EMMIX-GENE [9] and EMMIX-FDR [10] are software packages that apply this technique to cluster genes displaying similar expression patterns. However, these packages were not specifically developed to detect condition-specific expression, and therefore cannot be readily applied for this purpose on large datasets. Moreover, the method is not implemented in commonly used analysis platforms such as Bioconductor, making it difficult to integrate with additional analysis pipelines.

Two additional methods were recently developed with the specific aim of identifying condition-specific gene expression. First, a method called ROKU [11] implements Shannon's information theory entropy followed by an outlier detection method [12] to detect tissue specificity. This method is implemented in the Tissue Specific Genes Analysis (TSGA) R package [13]. It returns a list of conditions in which each gene is specifically expressed. Unfortunately, this method depends on a pre-defined set of ubiquitously expressed genes to model background expression levels - information that is generally not available prior to analysis. Furthermore, the TSGA method produces qualitative outputs - a gene is classified as either condition-specific or not without ranking genes or conditions - which makes the resulting lists difficult to prioritize for further analysis. Second, Vaquerizas *et al*. [2] previously used a propensity measure for a given gene to be expressed at a certain level in particular conditions relative to its expression across other conditions. The method provides a ranking of condition-specificity across samples. However, there is no control over the number of conditions in which a gene can be specific and there is no statistically meaningful threshold for specificity. Therefore, to our knowledge there is currently no straightforward and statistically robust method available to detect condition-specific gene expression.

Here we present a new method called SpeCond (for Specific Condition) to detect condition-specificity from a dataset of gene expression measurements. The method fits a normal mixture model to the expression profile of each gene, and identifies outlier conditions. We compare SpeCond against several alternative approaches using a gold standard dataset and demonstrate that SpeCond outperforms other methods. Finally, we apply the SpeCond approach to a subset of the Genome Novartis Foundation SymAtlas dataset [14], and identify specifically expressed genes from 32 human tissues samples. The method is freely available as an R package within the Bioconductor software project [15-17] at [18].

## Results

### SpeCond in a nutshell

**Figure 1:**
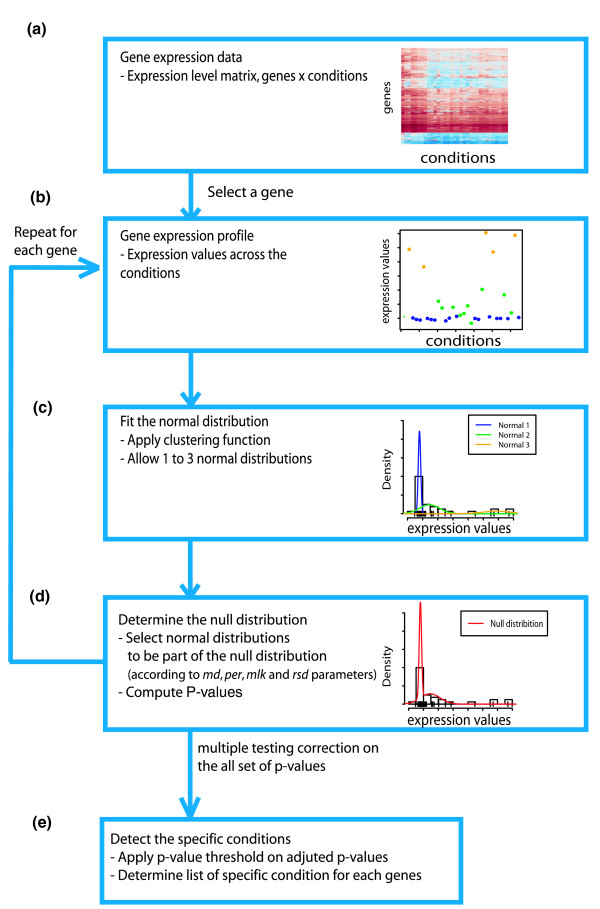
**SpeCond workflow**. **(a-e) **From a gene expression microarray dataset **(a)**, for every gene **(b)**, SpeCond uses a model of mixture of normal distributions **(c) **to determine the null distribution **(d) **and identifies the condition(s) in which the gene presents a statistically significant specific expression **(e)**.

Briefly, SpeCond examines the distribution of expression values for each gene in turn and then identifies outliers that indicate unusually high or low expression in specific conditions relative to others. It defines the background distribution for a gene across conditions using a normal mixture model. *P*-values are then calculated for the expression values of the gene across all conditions using the background distribution. After repeating the procedure for every gene in the dataset, SpeCond corrects all *P*-values for multiple testing. Finally, the method identifies condition-specific expression values for each gene using a *P*-value threshold (Figure 1). The different steps implemented in the method are described in detail below.

### Modeling the null distribution

Previous methods have modeled gene-expression values using a Gaussian distribution. However, most datasets do not fit this distribution well, as they often exhibit varying degrees of skewness [8]. To overcome this, we use a mixture model that fits between one and three normal distributions to the expression profile of a given gene (Figure 1c). This is achieved using the mclust package [19-21] in the R software environment [16,15]. The algorithm performs a hierarchical clustering of a mixture model of normal distributions via expectation-maximization. The best-fitting model is then selected using the Bayesian information criterium.

**Figure 2:**
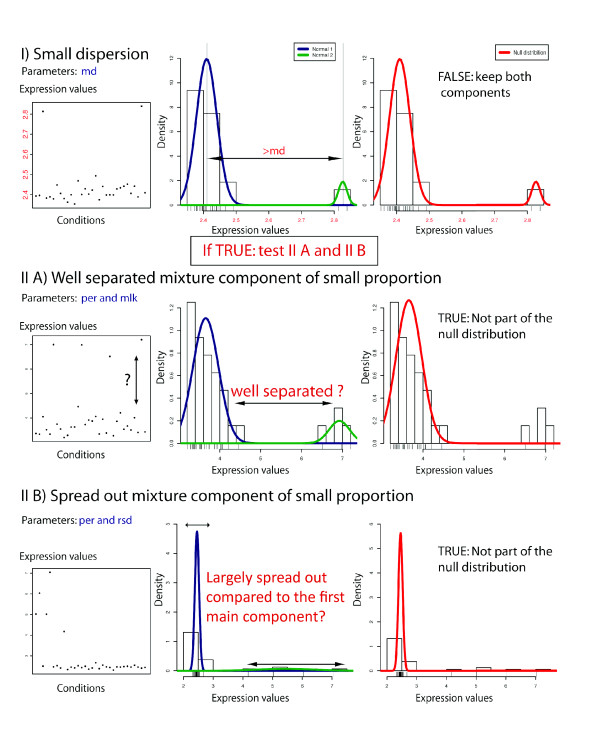
**Determination of the null distribution**. Three different conditions are evaluated in order to consider a normal component as part of the null distribution. (I) The median of the values from each component must have a difference larger than *md*. If this first condition is fulfilled, the procedure tests the following conditions. The normal component will not be part of the null distribution if: (II) the normal component is small and well separated, that is, the minimum of the absolute log-likelihood ratio of the expression values under the two components is larger than *mlk*; or (III) the normal component is small and largely spread out, that is, the standard deviation ratio is smaller than *rsd*.

In order to define the null distribution of a given gene (Figure 1d), we identify and exclude the mixture component(s) corresponding to outliers. First, we test whether the mixture component has a median value distinct enough from the median of the main component (test performed using the *md *parameter). If this is true, we then evaluate the following two possible scenarios (Figure 2): (i) whether the mixture component represents a small proportion of the data and is well separated from the main component; and (ii) whether the mixture component represents a small proportion of the data and has a large standard deviation compared with the main component. Mixture components that satisfy either of these criteria are likely to contain specific expression values and will therefore be excluded from the null distribution. Once all mixture components have been evaluated, the remaining components are combined using their means, standard deviations and relative weights. By default, if only a single component fits the data, its mean and standard deviation is used for the null distribution. As a result, our approach returns the optimal model for expression values after the identification of outliers.

### Identifying condition-specific expression values

Next, SpeCond computes a *P*-value for every expression value to determine whether a gene is specifically expressed. These *P*-values are based on the null distribution of each gene, and are computed as the sum of the *P*-values obtained from each mixture component, weighted by the proportion of the component in the mixture model. This procedure is applied to each gene in turn, and the overall set of *P*-values is corrected for multiple testing (Benjamini and Yekutieli method [22]).

Finally, a gene is determined to be specific if at least one adjusted *P*-value is below the specified threshold (pv parameter set to 0.05 by default). As a result, SpeCond classifies each gene as either displaying specific expression or not and returns the list of condition(s) in which it is specific (Figure 1e).

### User-defined parameters

SpeCond's behavior is determined by a set of user-defined parameters. These can be classified into three classes: (i) those controlling the implementation of the normal mixture model (λ and β); (ii) those used to decide which normal distributions are included in the final null distribution (*md*, *per*, *mlk *and *rsd*); and (iii) a *P*-value threshold to define a gene as being condition-specific (pv). A more detailed description of the parameters, including our choice for the default parameters, is given in Additional file 1.

### Comparison with other approaches

We chose the Genomics Institute of the Novartis Research Foundation (GNF) dataset [14] to evaluate the performance of our method. This dataset contains genome-wide expression profiles for 79 human tissues and cell lines. To avoid redundancy of tissue types within the dataset, we focused on 32 major healthy tissues and organs present in the dataset (Table 1). We first processed the data and determined the log2 expression level for each probe set in each condition as described in Additional file 1. We then applied SpeCond and two other alternative approaches, namely TSGA and the propensity method, to retrieve tissue-specific gene sets (see Additional file 1 for the choice of parameters).

**Table 1:** Numbers of tissue-specific genes for 32 human tissues

	Number of tissue-specific genes		
	
Tissue	Up-regulated	Down-regulated	Total
Whole brain	511	4	515
Whole blood	440	9	449
Testis	436	1	437
Fetal brain	406	4	410
Placenta	354	4	358
Liver	287	4	291
Skeletal muscle	279	16	295
Lung	278	1	279
Spinal cord	266	0	266
Fetal liver	261	0	261
Fetal lung	258	1	259
Thymus	249	0	249
Thyroid	244	1	245
Prostate	236	2	238
Smooth muscle	229	4	233
Heart	228	5	233
Bone marrow	227	0	227
Kidney	208	0	208
Uterus	199	1	200
Lymph node	194	1	195
Tonsil	179	0	179
Appendix	171	4	175
Pituitary	165	1	166
Trachea	162	0	162
Tongue	160	0	160
Pancreas	148	0	148
Skin	136	2	138
Adrenal gland	120	0	120
Fetal thyroid	119	1	120
Salivary gland	115	1	116
Adrenal cortex	85	0	85
Ovary	76	0	76

**Figure 3:**
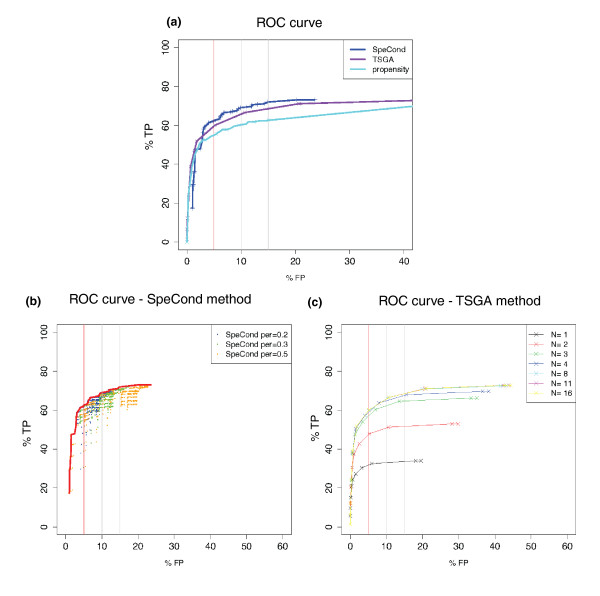
**Evaluation of the performance of the three methods**. **(a) **SpeCond (blue), TSGA (purple), propensity method (cyan). **(b,c) **Detailed parameter evaluation for the SpeCond and TSGA methods. (b) SpeCond: varying *mlk *(0 to 300) and *rsd *(0 to 2) parameters for the two steps with β = 6 and 0 and per = 0.1 and (0.2, 0.3, 0.5) for step 1 and step 2, respectively. The red line represents optimal parameter set for each false positive (FP) rate. **(c) **TSGA for the maximum number of specific tissue that can be detected N = 1, 2, 3, 4, 8, 11, 16, varying the H.critical value from 1 to 19.8. TP: true positive.

Using positive and negative gold standard sets containing previously defined specifically and ubiquitously expressed genes, respectively (Additional file 1), we computed receiver operating characteristic (ROC) curves to compare the performance of the three methods (Figure 3). Considering a 5% error rate, SpeCond achieved the best sensitivity of all methods (62%; Figure 3a). TSGA also showed good performance (60%), whereas the propensity method had lower sensitivity (55%).

We also performed a Gene Ontology (GO) enrichment analysis using the g:Profiler web-tool [23] and computed overall log-scores to compare the performance of each method from a biological perspective (Additional file 1). SpeCond and TSGA showed similar enrichment levels, outperforming the propensity method (log-scores = 18,316, 17,664, and 15,629 for SpeCond, TSGA and the propensity method, respectively). Therefore, overall, SpeCond displays better sensitivity and specificity than either of the other available methods.

### Detecting tissue specificity across the human genome

**Figure 4:**
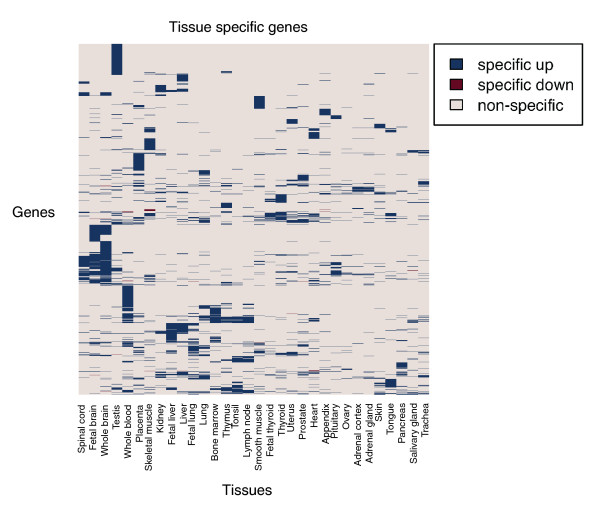
**Heatmap representation of the tissue specificity of each gene**. The specific behavior of every specific gene (y-axis) in every tissue (x-axis) is represented by a colored box: blue if the gene is specifically up-regulated in the tissue, red if the gene is specifically down-regulated in the tissue, and light grey if the gene does not present any specific expression for the tissue.

To demonstrate the use of our method, we examined the tissue-specific gene set returned by SpeCond when applied to the GNF dataset. We identified 2,673 genes as specific using the combination of parameters that achieved the best sensitivity at a 5% false positive rate (Additional file 2). Of these, 1,133 genes were detected in only one tissue and 1,540 genes were specifically expressed among several tissues (up to a maximum of 9 tissues). Figure 4 depicts a heatmap of tissue-specificity profiles for these genes. The large majority (approximately 99%) of genes that were specific were due to an up-regulation in a few tissues; interestingly, however, we also detected some genes that are specifically down-regulated compared to other tissues.

To assess the biological significance of the results obtained with SpeCond, we performed a GO enrichment analysis for each set of tissue-specific genes. For 28 out of the 32 analyzed tissues, we observed many expected molecular functions and pathways. For example, the GO terms 'contractile fiber' and 'heart morphogenesis' are enriched in heart, 'spermatogenesis' is specifically enriched in testis, and 'T cell activation' is enriched in the thymus. The remaining four tissues show a smaller number of specific genes, which did not allow the identification of significantly enriched functions among the specific genes.

Closer examination of the 287 liver-specific genes detected by SpeCond showed many genes that are important for liver functions, such as amino acid and fatty acid metabolic processes or gluconeogenesis. Among them are genes previously known to have liver-specific expression, such as *NR1I3*, a key regulator of xenobiotic and endobiotic metabolism [24], and *INSIG1*, which takes part in metabolic control [25]. In addition, we found genes that had not been originally assigned to have a liver-specific function. One example is *ATF5*, which is implicated in differentiation, proliferation and survival in different cell types but whose function in liver had not been annotated. The first indication of its function as a regulator of the hepatic stress response was recently published [26].

Another example is illustrated by the central nervous system. The brain, fetal brain and spinal cord present the largest list of tissue-specific genes (511 for brain, 406 for fetal brain and 266 for spinal cord; Table 1) and share 144 specific genes showing neural-related specific expression patterns. Functional profiling of tissue-specific genes shared by the three tissues revealed well-known nervous-tissue functions such as 'generation of neuron', 'axonogenesis', and 'synaptic transmission', as well as the neural cellular component 'neurofilament cytoskeleton'. In addition, we were able to identify *EAAT1 *(Excitatory amino acid transporter 1) as specific in the three tissues outlined above. This gene is known as a member of a family of high-affinity sodium-dependent transporter molecules that regulate neurotransmitter concentrations at the excitatory glutamatergic synapses of the mammalian central nervous system [27]. Further, we detected many genes with expression profiles specific for these tissues that have not been experimentally associated with any neural function in small-scale studies. Among these we found *ZNF365 *and *ZNF536*, two transcription factors previously reported to have brain- and spinal cord-specific expression [2].

### Bioconductor R package

**Figure 5:**
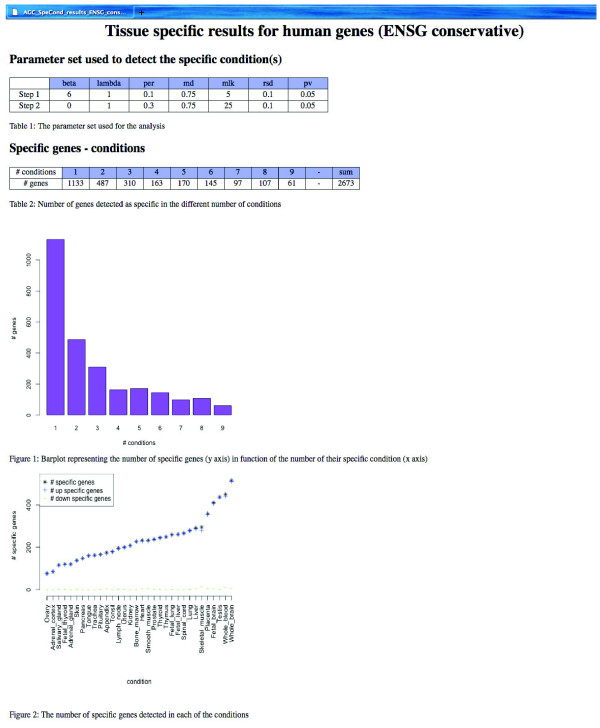
**SpeCond general HTML output (part 1)**. The HTML page displays a set of tables and figures: the parameters used in the analysis (first table), the number of genes detected as specific in different numbers of conditions (first figure and second table), and the numbers of specific genes (up-regulated, down-regulated and total) detected in each condition (second figure).

**Figure 6:**
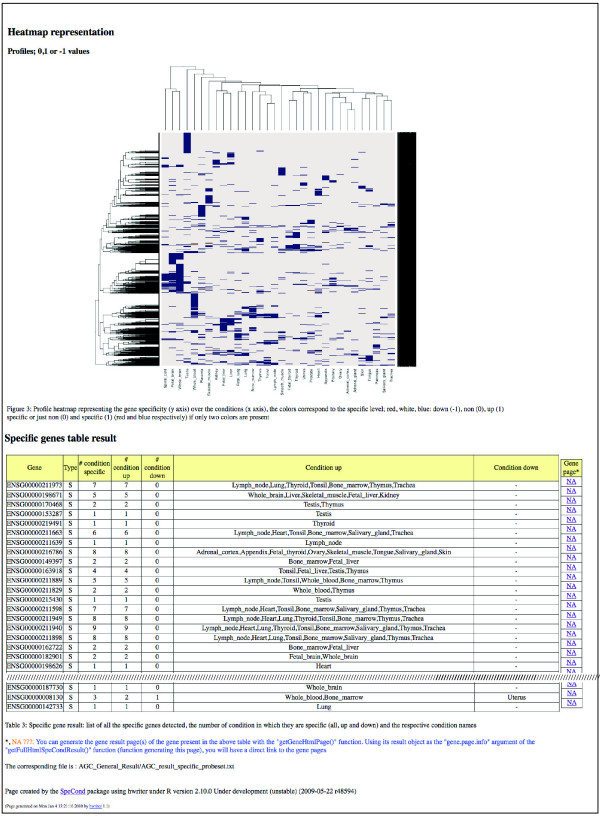
**SpeCond general HTML output (part 2)**. Following the tables and figures presented in Figure 5, the SpeCond general HTML page presents a heatmap of the tissue-specific genes (third figure) followed by a table containing all the tissue-specific genes with the numbers and tissues in which they are detected (up and down are separated). Links to an individual SpeCond specific HTML page, such as shown in Figure 7, are present in the rightmost column of the table if the pages have been previously generated by the user.

**Figure 7:**
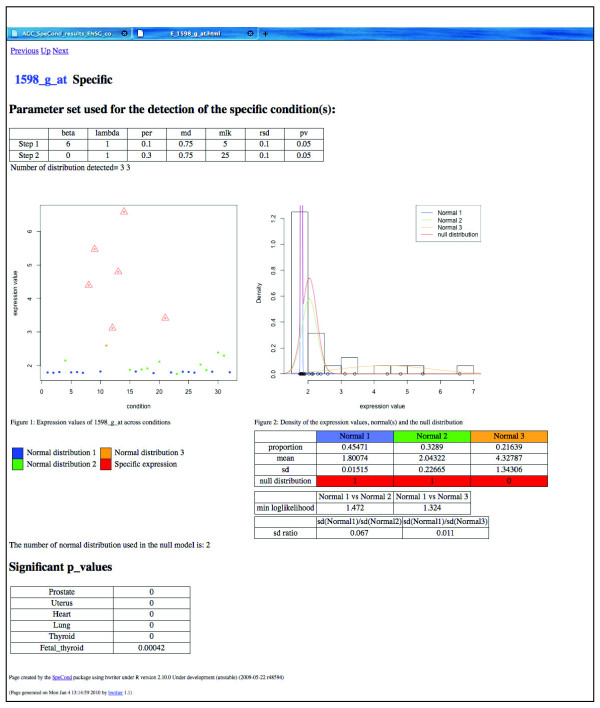
**Individual SpeCond specific HTML page; output for a specific probe set**. Example of the 1598_g_at probe set detected as specific in six tissues. The HTML page displays the probe set (or gene) name and a set of tables and figures: the parameters used in the analysis (top table), the expression profile (first figure), and the density curves of the mixture model fitting the expression values (normals 1, 2 and 3, in blue, green and yellow, respectively), as well as the null distribution (red) (second figure). The parameters of each normal distribution as well as the SpeCond parameter values are presented in the table below the second figure. Finally, the tissues in which the gene is detected as specific with their corresponding adjusted *P*-value are presented in the bottom table.

In order to provide easy access to the method, we developed SpeCond as an R package integrated within the Bioconductor software (freely available from [18]). The input to the software package is a matrix of normalized expression values in which rows correspond to genes or probe sets, and columns correspond to different conditions. The package returns different outputs: (i) R objects, (ii) text files that can be used for further analysis, and (iii) HTML pages. A general HTML results page provides an overall view of the condition-specific behavior for the entire dataset (Figures 5 and 6). Furthermore, an individual results page can also be generated for each gene (Figure 7; Additional file 3). The page displays an extensive set of figures illustrating the SpeCond analysis performed. Thanks to a large set of visualization functions for the results provided, the user can easily test different configurations of the parameters to evaluate which combination correctly corresponds to their particular dataset.

## Discussion

The widespread use of microarrays in biological research over the past few years has generated a flood of data characterizing gene expression across many tissues in different species [28]. Determining tissue- or condition-specific expression from these datasets is an important aspect of genomic analysis. Indeed, genes with a particularly high expression level in few conditions are likely to be involved in cell-specific functions; therefore, such genes could represent good candidates for tissue markers or drug targets. However, this detection is difficult to perform using traditional statistical techniques and few other methods were available.

Here we present SpeCond, a new statistical method to detect condition-specific expression from microarray data. We show that SpeCond is able to detect reliable tissue-specific genes and we evaluated its performance against alternative approaches. In all cases, SpeCond displayed higher sensitivity and a lower false discovery rate. Importantly, the SpeCond package is not a black box; the user is encouraged to test different parameter sets to find the best sets returning meaningful results according to relevant biological questions. Indeed, the large set of visualization tools allows the user to examine expression patterns in detail, to verify the fitting of the normal mixture distribution, as well as to easily compare the overall specific gene sets resulting from the use of different sets of parameters. In addition, the selection of inputted conditions can alter the results outputted by SpeCond; therefore, the user might consider applying standard clustering methods to identify the global variability in expression patterns among the different conditions, before manually selecting the most relevant conditions for the analysis.

A further advantage of SpeCond is its ability to generate ranked lists of genes based on their tissue-specific expression. The ability to classify genes in regard to their contribution to tissue-specificity should be helpful to experimentalists that wish to identify candidate genes for detailed follow-up studies. In addition, these ranked lists can be used in computational approaches, such as the examination of the organization of tissue-specific transcriptional networks or the putative annotation of unknown gene functions based on their expression pattern.

In the future, it will be very interesting to analyze RNA-seq data with the same purpose. However, the model will need to be modified, since a normal distribution-based model would not be the best to fit sequencing data. A negative binomial distribution as used in the DESeq method [29] is certainly more appropriate, and therefore a mixture of negative binomial distribution model would need to be created.

## Conclusions

SpeCond is a new statistical method to detect condition-specific expression from microarray data. SpeCond does not impose a single normal distribution to estimate the underlying distribution but computes an estimate of the null distribution using a normal mixture model. SpeCond is an ideal choice when no previous data about the organization of the system under study are available, as it is not assumed that the measured expression values follow a single normal distribution. Finally, SpeCond is immediately applicable to many datasets measuring gene expression, including the detection of tissue-specific alternative splicing, in any species.

## Abbreviations

GNF: Genomics Institute of the Novartis Research Foundation; GO: Gene Ontology; ROC: receiver operating characteristic; TSGA: Tissue Specific Genes Analysis.

## Competing interests

The authors declare that they have no competing interests.

## Authors' contributions

FMGC, JMV and NML conceived the study. All authors contributed intellectually to the development of the project. FMGC implemented the method and performed the analyses. FMGC, JMV and NML wrote the paper. All authors read and approved the final manuscript.

## Supplementary Material

Additional file 1 **Supplementary material**. The document file contains further information about data processing (ROC curve, GO analysis). Additionally, we provide a more detailed description of the SpeCond parameters. Click here for file

Additional file 2 **Table of human tissue-specific genes**. The table lists the 2,673 human genes detected as specific. For each of the 32 tissues (column) the gene has a value of 1 if detected as specifically expressed, -1 if detected as specifically repressed or 0 if it does not present specific expression in the tissue. Click here for file

Additional file 3 **Individual SpeCond specific HTML page; output for a specific probe set**. Example of the 121_at probe set detected as specific in two tissues. The HTML page displays the probe set (or gene) name and a set of tables and figures: the parameters used in the analysis (top table), the expression profile (first figure), and the density curves of the mixture model fitting the expression values (normals 1, 2 and 3, in blue, green and yellow, respectively), as well as the null distribution (red) (second figure). The parameters of each normal distribution as well as the SpeCond parameter values are presented in the table below the second figure. Finally, the tissues in which the gene is detected as specific with their corresponding adjusted *P*-value are presented in the bottom table. Click here for file
