# Supplementary material for: SpeCond: a method to detect condition-specific gene expression
Source: Genome Biol. 2011 Oct 18;12(10):R101. doi: 10.1186/gb-2011-12-10-r101 (PMC3333772; doi:10.1186/gb-2011-12-10-r101)
Supplement: Additional file 1 — Supplementary material. The document file contains further information about data processing (ROC curve, GO analysis). Additionally, we provide a more detailed description of the SpeCond parameters. [file gb-2011-12-10-r101-S1.PDF]

Supplementary material

SpeCond: a method to detect condition-specific gene expression

Florence MG Cavalli, Richard Bourgon, Wolfgang Huber, Juan M Vaquerizas, Nicholas M Luscombe

SpeCond parameters

Three main sets of parameters control SpeCond's behaviour: (i) those controlling the implementation of the normal mixture model; (ii) those used to decide which normal distributions are included in the final null distribution; and (iii) a *p*-value threshold to define a gene as being condition-specific.

The two parameters (λ and β) control how gene expression data are modelled as a mixture of normal distributions. λ (default value = 1) adjusts the weight of the effect of the number of parameters that need to be estimated when selecting the mixture model. As λ increases, the model penalises the inclusion of more parameters (i.e., more normal distributions). β (default value = 1) establishes a prior used to determine the variance of the normal distribution (see SpeCond vignette)

Four parameters (*md*, *per*, *mlk* and *rsd*; Figure 2) control the inclusion of the different mixture components in the final null distribution. *md* (default value = 0.75) is the minimum distance between median values of any pair of normal components. *per* (default value = 0.3) is the maximum proportion of conditions in which a gene can be detected as specifically expressed. *mlk* (default value = 25) is the minimum log-likelihood for a set of expression patterns given a pair of normal components. *rsd* (default value = 0.1) is the ratio of the standard deviations of a pair of a normal components.

A mixture component is excluded from the null distribution under the following circumstances: (i) when *md* is larger than the threshold and *per* and *rsd* are smaller than their respective thresholds —this scenario corresponds to normal components that are a small proportion of the data, separated from the main component and with large standard deviation; and (ii) when *per* is smaller than then threshold and *md* and *mlk* are larger than their respective thresholds —this scenario corresponds to normal components that are a small proportion of the data, well separated from the main component with little or non-existent overlap.

Finally, pv (default value = 0.05) determines the adjusted *p*-value threshold under which genes are classified as condition-specific.

It is worth noting that in order to consider multiple gene expression profiles, SpeCond uses a two-step selection procedure. In the first step SpeCond runs with a stringent *per* parameter and β ≠ 0 (step 1: λ= 1, β= 6, *md* = 0.75, *mlk* = 5, *rsd* = 0.1, *per* = 0.1 and pv = 0.05), aiming to detect condition-specific expression for genes with significantly high expression value (often happening in a single condition). In the

second step, SpeCond uses a stricter set of parameters (but with a *per* parameter allowing more conditions to be detected as specific) (step 2: $\lambda= 1$, $\beta= 0$, *md* = 0.75, *mlk* = 25, *rsd* = 0.1, *per* = 0.3 and pv = 0.05) to allow detection at a finer level of detail. A full description of the parameters can be found in the user guide document of the SpeCond package in Bioconductor at [http://www.bioconductor.org/packages/release/bioc/html/SpeCond.html].

It is important to note that the number of conditions present in a dataset has an important effect on the results. Due to the intrinsic nature of the analysis, enough conditions need to be measured to determine whether tissue-specific expression is biologically relevant, i.e. if a dataset does not cover enough conditions, it will not be clear whether a gene is truly only expressed in a single tissue. Moreover, due to the null distribution estimation, the detection becomes more powerful and robust as the number of conditions increases. Therefore, we recommend that researchers use SpeCond with at least ten conditions in order to obtain relevant results.

GNF data analysis

We chose the SymAtlas dataset [1] that contains genome-wide expression profiles for 79 human tissues and cell lines to evaluate the performance of our method. To avoid redundancy of tissue types within the dataset, which would affect the selection of specific genes, we focused on 32 major healthy tissues and organs present in the dataset (Table 1). We performed quality checks using the arrayQualityMetrics package [2] and processed the raw data using the three-step GCRMA algorithm as implemented in the Bioconductor project [3]. We then computed the mean of the log2 expression values of the two replicates for each tissue and used it as an expression value for a given probe set in a given tissue. We utilised the annotation available in the Ensembl database (Ensembl v52, [4]) to map 17,064 probesets to 11,713 Ensembl ID genes. Only probe sets that mapped uniquely to genes were used for this analysis.

We applied SpeCond to this dataset with the following parameters: (step 1: $\lambda = 1$, $\beta = 6$, *md* = 0.75, *mlk* = 5, *rsd* = 0.1, per = 0.1), step 2: $\lambda = 1$, $\beta = 0$, *md* = 0.75, *mlk* = 25, *rsd* = 0.1, per = 0.3 and pv = 0.05). Finally, in order to consider a gene as tissue-specific, we required that all associated probe sets were detected as specific in a given tissue. The combination of parameters was chosen to achieve the best sensitivity at a 5% false positive rate as measured using Receiver Operating Characteristic (ROC) curves (Figure 4). The results of the analysis are present in Additional file 2.

Gold standards

To evaluate the accuracy of SpeCond, we compared the results obtained with our method against other available approaches, namely TSGA and propensity.
To do so we computed ROC curves for each method using a gold standard set composed of ubiquitous and tissue-specific genes (Figure 4). For tissue-specific genes —our positive control— we computed the intersection of human tissue-specific genes from the Tissue-specific Gene Expression and Regulation (TiGER) database (based on EST data in 30 human tissues [5]) and those determined by Dezso and colleagues (expressed in one tissue among the 31 analysed in microarray expression analysis

[6]). Overall, our positive control group contained 3,984 probe sets in a total of 26 tissues, resulting in 5,595 gene-specific conditions. In order to obtain a set of ubiquitously expressed genes —our negative control— we merged two datasets: (i) the list of negative strand matching probe sets found in Affymetrix's HG-U133a array [7]; and (ii) the union of housekeeping genes detected in two independent studies [8,6]. In total our negative control contained 3,657 probe sets. Since these probe sets correspond either to genomic loci that should produce background signal —negative stand matching probe sets as they do not correspond to gene coding regions— or to genes that do not display tissue specificity across ~30 human tissues, we are confident that these lists represent an unbiased estimate of ubiquitous expression. However, it is worth noting that due to differences in origin, handing and sample preparation, the overlap and detection of tissue-specificity might not be exactly the same across all datasets.

ROC curve

As neither of the TSGA and propensity methods is able to detect tissue-specific repression, only genes detected by SpeCond showing tissue-specificity for higher expression were considered in this analysis.

To obtain the ROC curves for each method, we proceeded as described below. Briefly, for different values of the method's parameters, we obtained the tissue-specific genes per tissue then computed the percentage of true and false positive according to our gold standards.

- SpeCond method: we varied *mlk* from 0 to 300 and *rsd* from 0 to 2 for the two steps. Moreover β=6 and 0 and per=0.1 and (0.2, 0.3, 0.5) for step1 and step2, respectively. The best parameter set at each false positive percentage level was kept to draw the SpeCond ROC curve (red line in Figure 4, B and blue line in Figure 4, A).
- TSGA method: for a given maximum number of specific tissues that can be detected (N=1, 2, 3, 4, 8, 11, 16), we varied the H.critical parameter from 1 to 19.8. The best curve obtained corresponding to N=11 was used in the Figure 4, A (purple line).
- Propensity method: we varied the only parameter p that corresponds to propensity value threshold (for a given gene in a given condition) between 0 and 25 (i.e p=0, 0.25, 0.5, 0.75, 1, 1.25, 1.5, 1.75, 2, 25).

Go enrichment analysis

To perform a valid comparison of each method, we established a common threshold for false positive detection at 5% and adjusted the parameters of each method to provide the best sensitivity (number of true positives). These parameters were used to detect the set of tissue-specific genes on which the Gene Ontology (GO) analysis has been performed.

Given the set of genes detected as specific for each tissue, we performed a GO enrichment analysis using g:Profiler [9]. To measure the global performance of SpeCond, we computed a general log-score for each tissue as the sum of the

logarithms of significant p-values of the individual GO enrichments and compared it with log-scores obtained from random sets of genes [10].

To compare the performance of the three methods (SpeCond, TSGA and the propensity method), from a biological perspective, we computed a general log-score. To do so, we first performed GO enrichment analysis for the tissue-specific gene-sets returned by the three methods at 5% error rate (as above). Then the general log-score for each method was computed as the sum for the log-score obtain for each tissue.

Specific/selective detection

It is worth noting, that in the literature, some authors make a further distinction between specific and selective genes: specific genes are significantly differentially expressed in only one tissue whereas selective ones are differentially expressed in a small group, with the precise number of tissues often being left to individual choice. For this work, we refer to both categories as specific genes.

References

1. Su AI, Wiltshire T, Batalov S, Lapp H, Ching KA, Block D, Zhang J, Soden R, Hayakawa M, Kreiman G, Cooke MP, Walker JR, Hogenesch JB: **A gene atlas of the mouse and human protein-encoding transcriptomes**. *Proceedings of the National Academy of Sciences of the United States of America* 2004, **101**:6062–7.

2. Kauffmann A, Gentleman R, Huber W: **arrayQualityMetrics–a bioconductor package for quality assessment of microarray data**. *Bioinformatics* 2009, **25**:415–416.

3. Wu Z, Irizarry RA, Gentleman R, Martinez-Murillo F, Spencer F: **GCRMA A Model-Based Background Adjustment for Oligonucleotide Expression Arrays**. *Journal of the American Statistical Association* 2004, **99**:909.

4. Hubbard TJP, Aken BL, Ayling S, Ballester B, Beal K, Bragin E, Brent S, Chen Y, Clapham P, Clarke L, Coates G, Fairley S, Fitzgerald S, Fernandez-Banet J, Gordon L, Graf S, Haider S, Hammond M, Holland R, Howe K, Jenkinson a, Johnson N, Kahari a, Keefe D, Keenan S, Kinsella R, Kokocinski F, Kulesha E, Lawson D, Longden I, et al.: **Ensembl 2009.** *Nucleic acids research* 2009, **37**:D690–7.

5. Liu X, Yu X, Zack DJ, Zhu H, Qian J: **TiGER: a database for tissue-specific gene expression and regulation.** *BMC bioinformatics* 2008, **9**:271.

6. Dezso Z, Nikolsky Y, Sviridov E, Shi W, Serebriyskaya T, Dosymbekov D, Bugrim A, Rakhmatulin E, Brennan RJ, Guryanov A, Li K, Blake J, Samaha RR, Nikolskaya T: **A comprehensive functional analysis of tissue specificity of human gene expression.** *BMC biology* 2008, **6**:49.

7. Warren P, Taylor D, Martini PGV, Jackson J, Bienkowska J: **PANP - a New Method of Gene Detection on Oligonucleotide Expression Arrays**. *2007 IEEE 7th International Symposium on BioInformatics and BioEngineering* 2007, :108–115.

8. Eisenberg E, Levanon EY: **Human housekeeping genes are compact**. *Trends in Genetics* 2003, **19**:362–365.

9. Reimand J, Kull M, Peterson H, Hansen J, Vilo J: **g:Profiler–a web-based toolset for functional profiling of gene lists from large-scale experiments.** *Nucleic acids research* 2007, **35**:W193–200.

10. Reimand J, Vaquerizas JM, Todd AE, Vilo J, Luscombe NM: **Comprehensive reanalysis of transcription factor knockout expression data in Saccharomyces cerevisiae reveals many new targets**. *Nucleic Acids Res* 2010, **38**:4768-4777.
