# Supplementary material for: SpeCond: a method to detect condition-specific gene expression
Source: Genome Biol. 2011 Oct 18;12(10):R101. doi: 10.1186/gb-2011-12-10-r101 (PMC3333772; doi:10.1186/gb-2011-12-10-r101)
Supplement: Additional file 3 — Individual SpeCond specific HTML page; output for a specific probe set. Example of the 121_at probe set detected as specific in two tissues. The HTML page displays the probe set (or gene) name and a set of tables and figures: the parameters used in the analysis (top table), the expression profile (first figure), and the density curves of the mixture model fitting the expression values (normals 1, 2 and 3, in blue, green and yellow, respectively), as well as the null distribution (red) (second figure). The parameters of each normal distribution as well as the SpeCond parameter values are presented in the table below the second figure. Finally, the tissues in which the gene is detected as specific with their corresponding adjusted P-value are presented in the bottom table. [file gb-2011-12-10-r101-S3.PDF]

Additional file 3

Previous Up Next

121_at Specific

Parameter set used for the detection of the specific condition(s):

	beta	lambda	per	md	mlk	rsd	pv
Step 1	6	1	0.1	0.75	5	0.1	0.05
Step 2	0	1	0.3	0.75	25	0.1	0.05

Number of distribution detected= 2 2

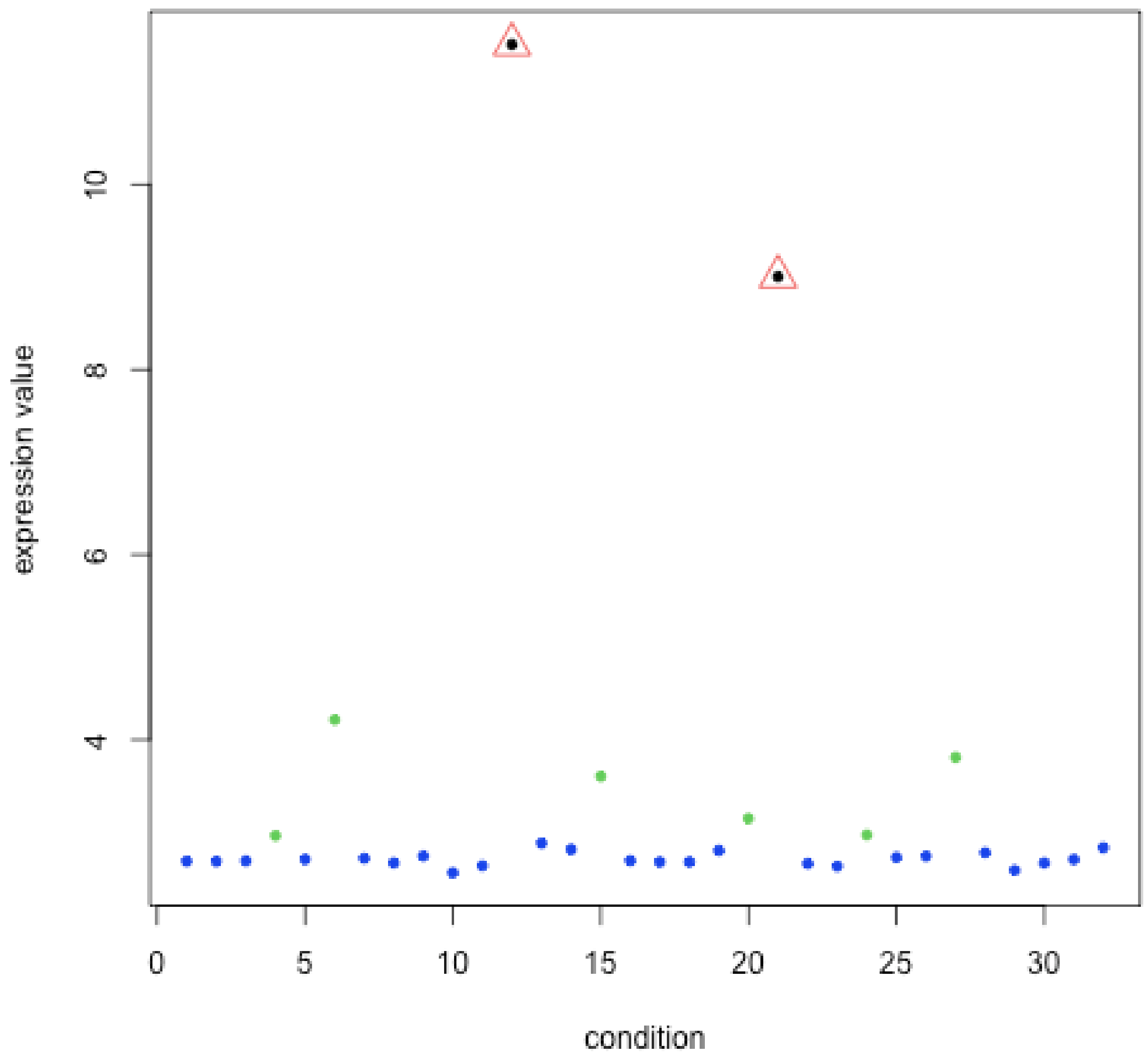

Figure 1: Expression values of 121_at across conditions

	Normal distribution 1		Specific expression
	Normal distribution 2		-

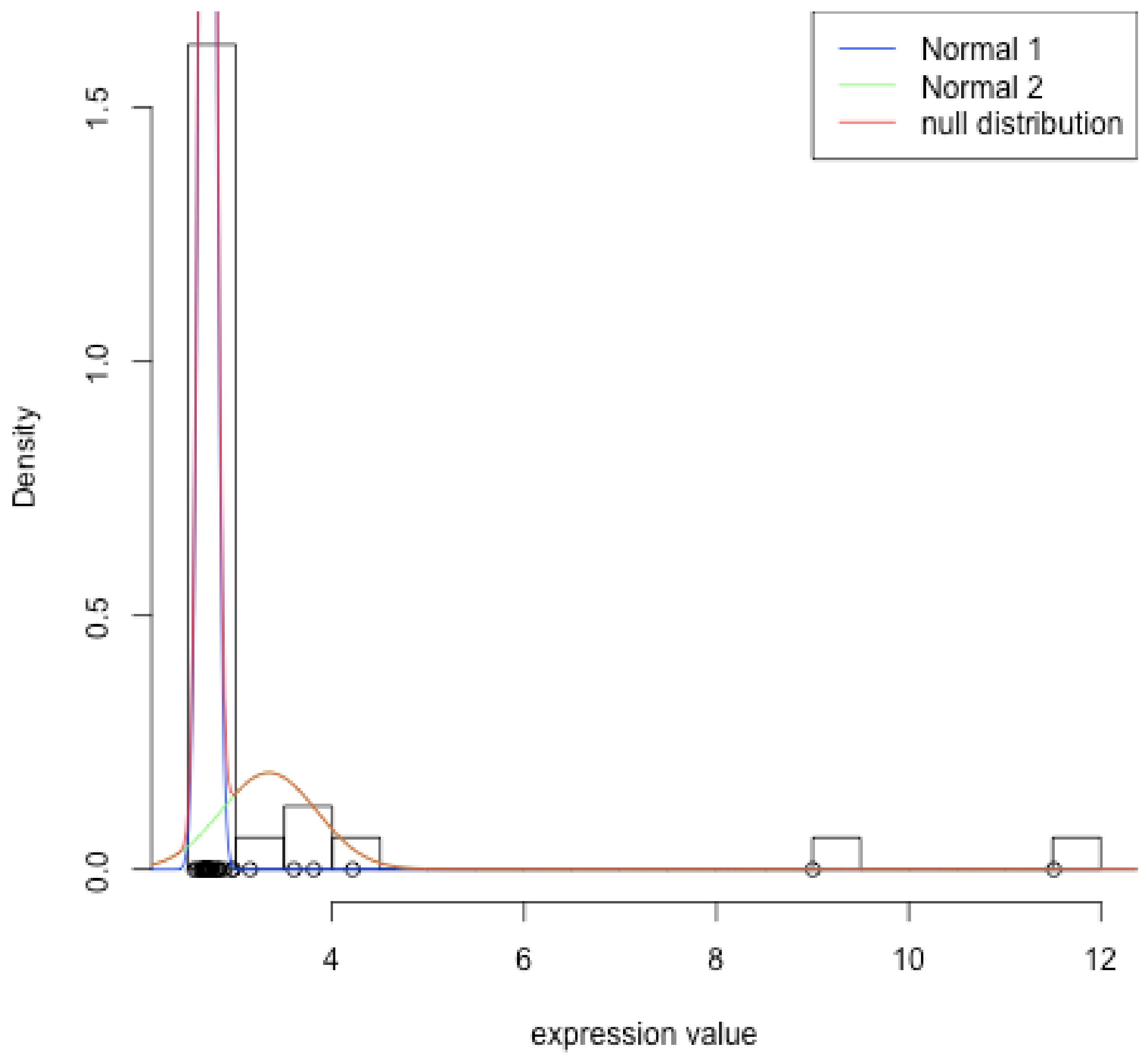

Figure 2: Density of the expression values, normal(s) and the null distribution

	Normal 1	Normal 2
proportion	0.76283	0.23717
mean	2.70573	3.34309
sd	0.07096	0.49735
null distribution	1	1

	Normal 1 vs Normal 2
min loglikelihood	0.718

	sd(Normal1)/sd(Normal2)
sd ratio	0.143

The number of normal distribution used in the null model is: 2

Significant p_values

Fetal_thyroid	0
Thyroid	0

Page created by the SpeCond package using hwriter under R version 2.10.0 Under development (unstable) (2009-05-22 r48594)

(Page generated on Mon Jan 4 13:14:57 2010 by hwriter 1.1)
